# Characterization and critical appraisal of physiotherapy intervention research in Nigeria: a systematic review

**DOI:** 10.1186/s12891-023-06986-7

**Published:** 2024-01-02

**Authors:** Martins Nweke, Emeriewen Ejiroghene, Henrietta O. Fawole, Nombeko Mshunqane

**Affiliations:** 1 Department of Physiotherapy, Faculty of Health Sciences, University of Pretoria, Pretoria, South Africa; 2 Department of Physiotherapy, School of Basic Medical Sciences, College of Medical Sciences, University of Benin, Benin, Edo State Nigeria

**Keywords:** Physiotherapy, Clinical research, Characterization, Appraisal, Nigeria

## Abstract

**Objectives:**

Clinical research is the bedrock of clinical innovation, education and practice. We characterized and critically appraised physiotherapy clinical research to avoid implementing misleading research findings into practice and to task the Nigerian physiotherapy societies on responsible conduct of clinical research.

**Methods:**

This is a systematic review of articles published in English between 2009 and 2023. We started with 2009 because at least few Nigerian Physiotherapy school had commenced postgraduate (research) training by then. We searched Pubmed, Medline, Cumulative Index to Nursing and Allied Health Literature, Academic Search Complete, PsycINFO and African Journal Online, and reference lists of relevant articles. We Data were selected and extracted according to predesigned eligibility criteria and using a standardized data extraction table. Where appropriate, the Pedro and Cochrane ROBINS1 were used to examine the risk of bias.

**Results:**

A total of 76 Nigerian studies were included in this study. The mean age of the study participants was 46.7 ± 8.6 years. Approximately, 45% of the participants were males. Of the clinical experiments, the randomized controlled trial (RCT) was the most common design (87.5%). Musculoskeletal conditions (39.3%) were the most studied disorder. Approximately 86% of the RCT had studies possessed fair to good quality. Interventions constituted exercise therapy (76.3%), manual therapy (8.5%) and electrotherapy (8.5%). More than half (67.8%) of the studies recorded medium to large effect sizes. A fair proportion (48.2%) of the studies had a confounding-by-indication bias. Approximately 43% of the clinical experiments were underpowered, and a few studies conducted normality tests (10.9%) and intention-to-treat analysis (37.5%).

**Conclusions:**

RCT is the most frequent clinical experiment, with majority of them possessing fair to good quality. The most important flaws include improper computation of sample size, statistical analysis, absent intention-to-treat approach, among others. The magnitude of effects of Physiotherapy interventions varies from nil effect to large effect. Musculoskeletal condition is the most prevalent disorder and exercise is the most important intervention in Nigerian physiotherapy practice.

**Trial registration:**

We registered the protocol with PROSPERO. The registration number: CRD42021228514.

**Supplementary Information:**

The online version contains supplementary material available at 10.1186/s12891-023-06986-7.

## Introduction

Clinical research is the bedrock of clinical innovation, education and practice in both developed and developing nations [1]. Without it, there can be no advancement in health and clinical practice, and philosophy, opinions, mere assumptions and quackery will occupy the front rows in the care for the whole man [2]. The competitive search for the Coronavirus-19 vaccine through clinical inquiries illustrates the typical role of clinical research in health development and innovation [3]. The role of clinical research is multifaceted and cannot be overemphasized. It provides information about disease trends and risk factors, outcomes of treatment, public health intervention, functional abilities, patterns of care, health care costs amongst others [4]. Health research has led to the development of new therapies, and remarkable improvement in health care and public health [5]. They form the primary means by which a nation’s healthcare goals are achieved and improved [6]. Interestingly**, **no therapy, testing procedure or clinical device is deemed safe and effective for public consumption except by evidence emerging from clinical research [7]. The practice of physiotherapy in Nigeria is majorly informed by evidence emerging from high-income countries, with little inputs from the local context to validate and contextualize evidence obtained in high income countries. This is largely due to poor research culture among Nigeria clinical physiotherapists, and lack of postgraduate physiotherapy education until 2009 when a few Nigeria Universities commenced postgraduate training in Physiotherapy [8].

Now, physiotherapy education is over 50 years in Nigeria [9], however, evidence emerging from an a multi-center study reveals a low level of participation in clinical research among Nigerian Physiotherapists [10], with outputs of these research being negatively skewed towards non-clinical observational studies [11]. While one cannot dispute the utter relevance of non-clinical research, the need for clinical and intervention research towards an effective Physiotherapy practice is essential and cannot be overemphasized [6]. The quality of Nigerian-based clinical research, reportedly, leaves much to be desired [12]. This possesses implications for both implementations science and clinical practice in a country where Physiotherapy is fast becoming a routine treatment. The overt dependency on research innovations and patency obtained in developed nations as well as poor research funding constitutes a hindrance to the development of clinical Physiotherapy in the Nigerian context. In a country hosting over 50 PhD students every four to five years, one would expect a fair degree of innovations and growth of the Physiotherapy profession [13], with the contextualization of practice to suit specific local needs and scarce resources. Furthermore, as at 2016, there were at least 114 academics in physiotherapy departments across the country, with 36 and 69 of them possessing MSc and PhD respectively [14]. However, clinical physiotherapy practice is yet to reap of this investment, and this may be due to paucity of thoroughly informed and designed interventional studies, lack of implementation of research findings and poor research funding which remain the major challenges in low and middle income contries [12, 15]. As part of the preliminary strategy directed at advancing clinical physiotherapy education and practice in Nigeria, this review aims to characterize and critically appraise physiotherapy intervention research in Nigeria.

## Methods

### Protocol and registration

This systematic review followed the Preferred Reporting Items for Systematic Reviews (PRISMA) checklist, and supplemented by the Joana Briggs Institute Scoping Review Guidelines [16]. The protocol is registered with PROSPERO, ID- CRD42021228514. We have made the following amendment to the review protocol since registration: the review aim has been recast from “to investigate the types & methodological quality of clinical physiotherapy research carried among Nigerian population” to “to characterize and critically appraise clinical interventional research in Nigeria, and to set forth recommendations for relevant stakeholders and policymakers”. Also, we discovered that Pedro and ROBINS 1 will be more appropriate compared to the mixed method appraisal tool specified in the registered study protocol.

### Eligibility criteria

“All articles were independently reviewed by two authors to determine their eligibility based on the following eligibility criteria: i). studies involving a Nigerian patient population; ii). studies examining the effect of a physiotherapeutic intervention in a Nigerian patient population regardless of whether or not a comparator was employed; iii). trials that included a pharmaceutical comparator but provided enough information to determine the physiotherapy intervention modality's independent effect; iv). outcomes constituted study characteristics (including design, sample size, intervention and disease studied), methodological quality, study effect size and power; v). articles written and published in English between 2009 and 2023; vi). studies were included irrespective of setting (primary, secondary, tertiary health care, private physiotherapy clinics/gym centers)” [16]. We excluded studies conducted before 2009, studies in which it is difficult to ascertain the stand-alone effect of the physiotherapeutic intervention employed or case reports, to enhance comparability.

### Information source

In line with the registered review protocol, we searched for original research articles and systematic reviews using PubMed, Cochrane Library, MEDLINE, CINAHL, Academic Search Complete, PsycINFO and African Journal Online. Furthermore, we searched the reference lists of relevant articles for additional peer-reviewed articles.

### Search

Initially, we harvested keywords and MeSH terms from key articles. We then analysed the keywords and index terms used to describe the articles. We conducted a pilot search using these terms in PubMed. We refined the search terms to obtain the most sensitive and specific strategy (See Additional file 1: Appendix 1). A third search using the piloted terms was conducted across all selected databases. The earliest search was carried in January 2021 while the latest search was done using AJOL in April 2023.

### Study selection

**Fig. 1:**
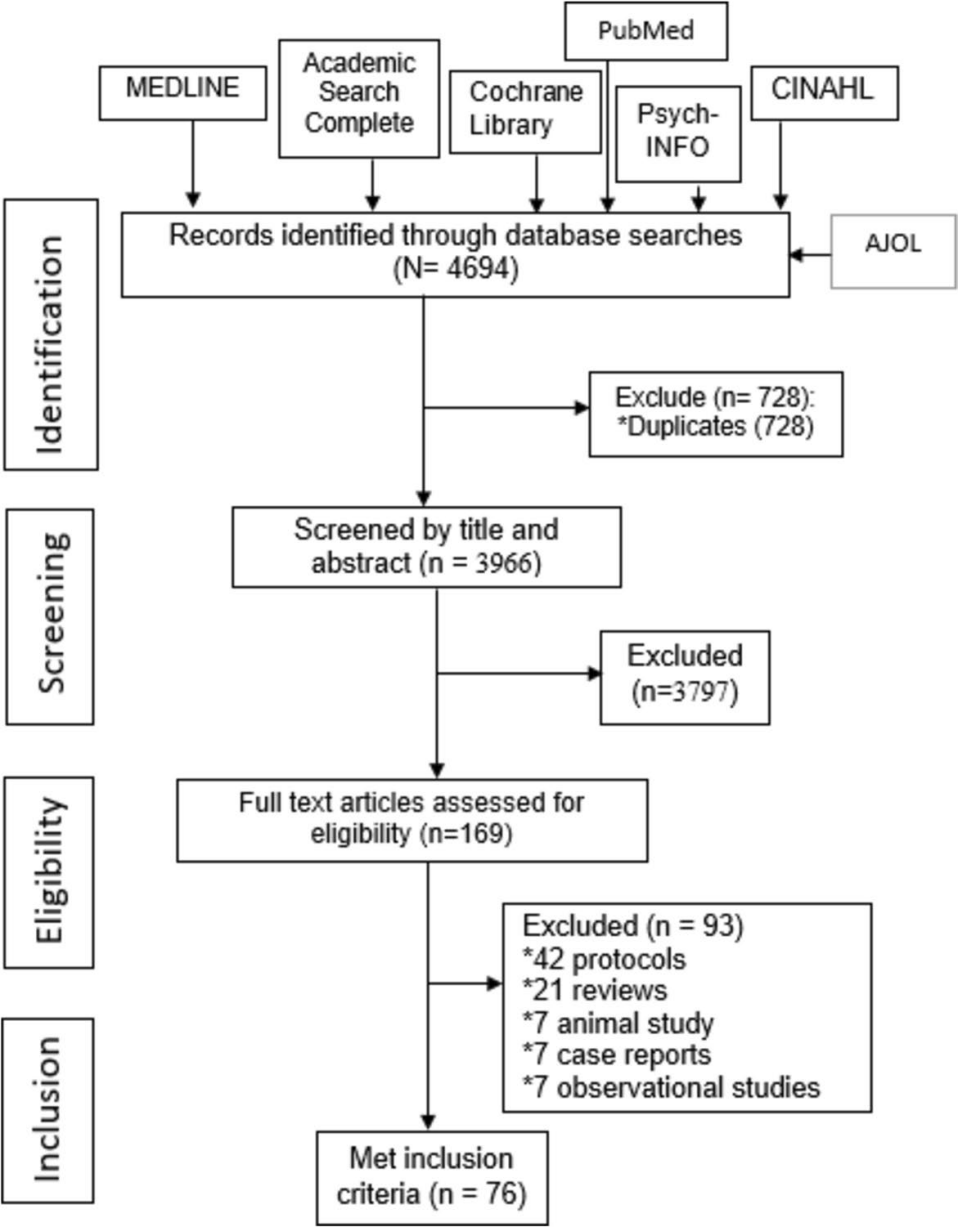
PRISMA flow diagram for the systematic review on characterization and appraisal of physiotherapy intervention research in Nigeria (2009–2020)

We exported our search results directly into EndNote 8, where duplicate articles were removed. Once all the duplicate articles were removed, a reviewer screened the titles and selected articles that met the inclusion criteria. EE reviewed the full-text articles and excluded articles that did not meet the eligibility criteria under the supervision of NM. We screened the reference lists of relevant articles to identify additional studies. We did not restrict studies based on target population, setting, language. We only included studies published after 2009. We only included studies published in English. We excluded case reports to enhance comparability. The PRISMA diagram details the flow of studies throughout the selection process, along with reasons for excluding articles (Fig. 1).

### Data collection process

In this review, data were extracted by EE and validated by NM. From each article, we extracted primary data including study characteristics (design, intervention and disease) and study quality. We also extracted data necessary to estimate the study effect size and post-hoc study power. We also recorded article information including author, title, population, sample size, sampling techniques, geopolitical region and summary of findings. Data were extracted using a custom spreadsheet. We contacted a study author to ask for full-text details.

### Data items

Primary data sought included study characteristics (design, sample size, intervention utilised, and disease condition), and study quality. Secondary data were age, sex, mean difference and summary of findings.

### Risk of bias assessment and quality appraisal

We undertook quality appraisal using the Pedro Risk of Bias Assessment Tool Scale and Cochrane ROBINS-1, depending on the design. The Pedro scale is a specialized and reliable methodological evaluation instrument for physiotherapy RCTs [17]. Only when a requirement is met is a point awarded. The reliability of Pedro scale item evaluations ranged from "fair" to "significant," while the entire Pedro score's reliability was "fair" to "good" [17]. The measure appears to be reliable enough to be used in systematic evaluations of RCTs in physical therapy [17]. Following we defined poor study quality as 0–3, fair as 4–5, good as 6–8 and excellent as 9–10 [18]. Similarly, the Cochrane ROBINS-I is the most commonly recommended tool for non-randomized clinical experiments [19]. ROBINS-I tool was used to measure the risk of bias in the methodology aspects of included non-randomized clinical studies. Studies’ risk of bias was considered low, moderate, serious or critical based on bias in these seven domains: confounding, participant selection, intervention, measurement, deviation from intended intervention, missing data, outcome measurement and selection of reported result. A study were considered to have low risk of bias if all the domains were ranked low; to have moderate risk of bias if the domains were ranked low or moderate; to have serious risk of bias if at least one domain was ranked serious; and to have critical risk of bias, if at least one domain was ranked critical [19].

### Summary measure and data synthesis

We obtained mean difference from studies and computed effect size per study, in line with Borenstein et al. [20] and Lenhard [21]. Post-hoc power was computed using the G-power software. Alpha was set at 0.05. For confounding-by-indication, we assessed studies for analysis of baseline comparison for socio-demographic and the key outcome. The assessment was scored over 5, 6 or 7 depending on the disorder. Age, gender, BMI and occupation (rarely) were the prevalent socio-demographics assessed (Table 1).

**Table 1:** Determination of confounding-by-indication bias

S/N	Disorder	Confounds	Pass-score including KO
1	Breast cancer	Age, medication, BMI, KO	2/4
2	Cerebral palsy	Age, severity, KO	2/3
3	COPD	Age, gender, respiratory rate, KO	2/4
4	Diabetes	Age, BMI, gender, KO	2/4
5	Heart failure	Age, gender, Heart rate, KO	2/4
6	HIV	Age, gender, viral load/CD4 counts, adherence to drugs, depression, KO	3/6
7	Hypertension	Age, gender, BMI, diabetes, KO	2/5
8	LBP	Age, gender, BMI, occupation, KO	2/5
9	Lumbar rad	Age, gender, BMI, occupation, KO	2/5
10	Menopause	Age, gender, BMI, peri/post variations, KO	2/5
11	Obesity	Age, gender, BMI, KO	2/4
12	Osteoarthritis	Weight, BMI, age, gender, occupation, KO	3/6
13	SCA	Age, gender, BMI, baseline Hb, KO	2/5
14	Stroke	Age, gender, BMI, severity, time, type of stroke, laterality	3/6
15	NS- Neck pain	Age, gender, BMI, occupation, KO	2/5
16	Pregnancy	Maternal age, trimester, febrile illness, intra-partner violence, maternal hypertension, KO	3/6
17	Piriformis syndrome	Age, gender, occupation, BMI, KO	3/5
18	Breast engorgement	Maternal age, parity, gravidium, education, KO	2/5
19	HIV-associated neurocognitive disorder (HAND)	Alcohol, substance abuse, depression, psychotic disorder, focal neurologic deficit, income, KO	4/7

*KO* Key outcome, *COPD* Chronic obstructive pulmonary disease, *BMI* Body mass index, *HIV* Human immunodeficiency virus, *Hb* Hemoglobin, *LBP* Low back pain, *SCA* Sickle Cell Anemia, *NS* Non specific

## Results

### Study selection and characteristics

We identified 2800 records in the first search of 7 databases. After de-duplication, 1990 records remained. After screening all the titles and abstracts, we excluded 1858 irrelevant records, leaving 132 records for full-text review. Of the 132 full-texts, 86 publications were excluded leaving 56 eligible articles. In the update search, we accessed two databases namely PubMed and African Journal Online. A total of 1807 were retrieved of which 1770 were excluded following title and abstract screening leaving 37 articles for full text screening. Of the 37 articles, 20 were eligible and included. Overall, our review included 76 articles involving 5733 participants from the country (Fig. 1 and Table 1). The included studies were published between 2010 and 2021.

### Participants’ characteristics

Seventy studies reported age, with mean age of 46.6 ± 8.1 years and 46.8 ± 9.2 years for experimental group and control groups, respectively. Forty-five studies reported gender, with male to female ratio for the experimental 47%:53% and 43%: 57% for the control group. Most of the studies did not report educational backgrounds but amongst studies (five) that did, the average percentage of participants with at least secondary education was 76.8% and 78.4% for experimental and control groups, respectively while 65.2% and 28.8% represent the number with primary or no formal education experiment and control groups, respectively (Additional file 1: Appendix 2).

### Quality appraisal

Of the 76 included studies, we assessed the quality of the 64 RCTs using Pedro. Of the 64 studies, 15 (23.4%) studies possessed fair quality, 40 (62.5%) possessed good quality and 9 (14.1) possessed excellent quality (Additional file 1: Appendix 3 Table 1). All the twelve non-randomised experiments were assessed using ROBINS-1 and 10 (83.3%) had low risk of bias (Additional file 1: Appendix 3 Table 2).

### Disorders, interventions and outcomes

A total of 19 disorders were studied. These include non-specific low back pain 19 (25%), type 2 diabetes mellitus (DM) 9 (11.8%), hypertension 9 (11.8%), osteoarthritis 9 (11.8%), and HIV infection 9 (11.8%), among others. The interventions utilized were exercises 58 (76.3%), manual therapy 9 (11.8%), electrotherapy 10 (13.2%), etc. Types of exercise were stabilization exercises, therapeutic exercise, aerobic exercises, mobilization exercise, resistance exercise, aquatic exercise, walking, McKenzie back exercise, isometric handgrip exercise etc. The three most studied key outcomes were pain intensity 26 (34.2%), blood pressure 11 (14.5%) and blood glucose 5 (6.6%) (Table 2).

**Table 2:** Effect size, and statistical power of the included studies

Author	Intervention for outcome(tool) in condition	*P*-values	Effect size	Power (%)	Remark
Abass et al. [22]	Lumbar stabilization for PI in chronic LBP	0.236	0.082	48	X
Abdulahi et al. [23]	CIMT improving MF in stroke	> 0.05﻿*	0.348	46	X
Abdullahi et al. [24]	CIMT for motor impairment in stroke	0.166	0.37	28	X
Adeniyi et al. [25]	Exercise improving for SBP in Type 2 DM	0.030﻿*	IN		-
Adeniyi et al. [26]	Exercise for pain in Type 2 DM	< 0.05*	IN		-
Adepoju et al. [27]	Exercise improving balance in CP	0.339	0.57	58	X
Ahmed et al. [28]	Stabilization exercise & mzl energy tech for pain in low back pain	< 0.001*	0.7385	59	X
Ajiboye et al. [29]	Exercise improving walking capacity in CHF (biventricular)	*p* < 0.05﻿*	1.28	100	√
Akinola et al. [24]	Exercise improving GMF in spastic CP	0.001*	0.236	9	X
Akodu & Akindutire [30]	Exercise for sleep disturbance in LBP (NS)	0.030﻿*	0.6	100	√
Akodu et al. [31]	Stabilization and pilate exercise for pain in LBP	0.001﻿*	1.239	96	√
Aliyu et al. [32]	Exercise for pain in LBP (NS)	0.6	0.0078	85	√
Asogwa et al. [33]	Progressive resistance exercise for CD4 count in HIV patients	< 0.001*	0.82	87	√
Aweto et al. [34]	Exercise for depression score in PL HIV	0.925	0.36	100	√
Aweto et al. [35]	Incentive spirometry for FVC in Type 2 DM	0.925	0.071	6	X
Aweto et al. [36]	Incentive spirometer for FEV in asthma	0.001﻿*	0.441	73	X
Bello & Adeniyi [37]	Exercise for pain intensity (VASB) in LBP	0.020﻿*	1.93	100	√
Bello et al. [38]	Manual therapy for FVC in lumbar radiculopathy	0.000﻿*	0.26	46	X
Bolarinde et al. [39]	Exercise for PI in LBP	< 0.001*	0.023	60	X
Danazumi et al. [40]	Integrated neuromuscular inhibition tech for pain in piriformis syndrome	< 0.001*	5.433	100	√
Danazumi et al. [41]	Chain Exercise & kinesiotaping for pain in OA	0.011﻿*	2.116	100	√
Ezema et al. [42]	Exercise for FBS in Type 2 DM	0.001*	0.59	100	√
Ezema et al. [43]	Exercise for SBP in PLHIV	<0.010	2.74	100	√
Ezema et al. [44]	Exercise for FBS in type 2 DM	< 0.01*	-1.281	98	√
Fadupin & Akinola [45]	Exercise for FBS in Type 2 DM	< 0.05*	0.16	100	√
Fayehun et al. [46]	Walking prescription for HBA1c in Type 2 DM	0.015﻿*	0.035	5	X
Habibu & Hanif [47]	Non-thermal US for breast engorgement	0.153	0.132	8	X
Ibrahim et al. [48]	Motor control exercise for pain in low back pain	< 0.05	0.767	87	√
Idowu & Adeniyi [49]	Graded activity with monitoring for PI in LBP and Type 2 DM	< 0.001﻿*	1.94	99	√
Ige et al. [50]	Pulmonary rehab for FEV1 in COPD	< 0.050*	2.518	100	√
Jegede et al. [51]	Exercise for WR in obese individuals	< 0.001﻿*	1.17	99	√
John et al. [52]	Exercise for SBP in PL HIV	0.001*	0.12	100	√
Johnson et al. [53]	McKenzie for PI in LBP	0.02*	1.18	79	X
Kaka et al. [54]	Exercise for VAS in NS-neck pain	0.001*	1.267	100	√
Lamina & Okoye [55]	Exercise for lipid profile in hypertension	0.001*	0.007	57	X
Lamina & Okoye [56]	Moderate intensity training for SBP in hypertension	0.001*	1.22	100	√
Lamina & Okoye [57]	Exercise for SBP in hypertension	0.001*	0.67	100	√
Lamina & Okoye [58]	Interval training program for SBP in hypertension	0.001*	0.27	100	√
Lamina et al. [59]	Shortwave for pain for pain in PID	< 0.001*	1.746	99	√
Lamina et al. [60]	Exercise for SBP in hypertension	0.001﻿*	1.04	100	√
Maduagwu et al. [61]	Exercise for QoL in HIV seropositive	0.917	0.023	6	X
Maduagwu et al. [62]	Exercise for CD4 cells on in HIV	0.002*	0.82	90	√
Maharaj & Nuhu [63]	Exercise for FBS in type 2 DM	0.002*	0.122	21	X
Maruf et al. [64]	Aerobic dance for lipid (LDL-C) IN HTN patients	< 0.05	-0.092	79	X
Maruf et al. [65]	Exercise for BP in hypertension	0.075	0.39	74	X
Maruf et al. [66]	Exercise for SBP in hypertensive patients	0.001﻿*	0.751	98	√
Mbada et al. [67]	McKenzie for DBEE in LBP	0.001﻿*	0.27	100	√
Mbada et al. [68]	Exercise for FAB in LBP	0.154	0.076	9	X
Nweke et al. [69]	Aerobic exercise (Cycle ergometer) for HAND	< 0.05﻿*	0.261	20	X
Nweke et al. [70]	Aerobic exercise (Cycle ergometer) for QoL in HAND	0.001*	3.156	100	√
Odebiyi et al. [71]	Exercise and massage for QoL of cancer patient	0.001*	0.39	100	√
Odole & Ojo [72]	Telephone based therapy for PI on OA	0.001﻿*	0.16	100	√
Odunaiya et al. [73]	Aerobic exercise for depression in HIV patients	< 0.05*	2.091	100	√
Ogbutor et al. [74]	Exercise for SBP in pre-hypertensives	1.653	1.5	100	√
Ogwumike et al. [75]	2 Exercises for Adiposity in peri- and post-menopausal	0.05	0.959	23	X
Ojeniweh et al. [76]	Infrared for pain in low back pain	> 0.05﻿*	-0.173	9	X
Ojoawo & Olabode [77]	TOP and traction for NDI in cervical radiculopathy	0.889	0.125	42	X
Ojoawo et al. [78]	Cervical traction for pain in cervical radiculopathy	< 0.05*	-1.218	98	√
Ojoawo et al. [79]	Exercise for PI in LBP	1.000	0.052	5	X
Ojoawo et al. [80]	US and massage for PI in LBP	0.001*	0.57	99	√
Ojoawo et al. [81]	TOP for PI in cervical radiculopathy	< 0.05*	0.53	85	√
Ojoawo et al. [82]	VOP for RMDQ in chronic LBP	0.692	0.549	68	X
Ojoawo et al. [83]	Exercise for VAS in knee OA	< 0.01*	0.386	87	√
Okonkwo et al. [84]	Balance training for balance in cognitively-impaired stroke patients	< 0.05*	3.37	100	√
Okonkwo et al. [85]	TENS for PI in injection sciatic pain	0.001*	0.16	13	X
Olagbegi et al. [86]	Exercise for SQMS in OA	> 0.05*	0.07	100	√
Olagbegi et al. [87]	Exercise for ADL in OA	< 0.001﻿*	0.737	100	√
Onigbinde et al. [88]	Wobble board exercises & dynamic balance for static balance in stroke patients	0.001*	1.213	63	X
Onigbinde et al. [89]	IQS for RMx in OA	0.001﻿*	0.644	89	√
Onigbinde et al. [90]	SWD for VAS in OA	0.03*	0.747	42	X
Onuwe et al. [91]	Double modality for PP in MSK injuries	< 0.05﻿*	0.293	24	X
Onwunzo et al. [92]	Isometric strengthening exercise for pain in OA	< 0.001﻿*	-4.907	100	√
Sarafadeen et al. [93]	Stabilization exercise for pain in LBP	< 0.001*	-4.207	100	√
Sokunbi et al. [94]	TENS for sleep quality in pregnant women	0.717	0.196	10	X
Tella et al. [95]	TENS for tactile acuity in LBP	< 0.05*	0.352	15	X
Usman et al. [96]	Combination therapy for pain in OA	0.001*	0.34	100	√

*: significant at α = 0.05; X: finding is probably not a reflection of true effect; √: finding is probably a reflection of true effect; *IN* Information not available, *DM* Diabetes mellitus, *CP* Cerebral palsy, *CIMT* Constraint-induced movement therapy, *CHF* Congestive heart failure, *WR* Weight reduction, *LBP (NS)* Low back pain (non-specific), *SBP* Systolic blood pressure, *Type 2 DM* Type 2 diabetes mellitus, *(G) MF* (Gross) motor function, *CHF* Chronic heart failure, *(PL) HIV* (People living with) Human Immuno-deficiency virus, *FVC* Forced vital capacity, *VAS (B)* Visual analogue scale (back), *PI* Pain intensity, *FBS* Fasting blood sugar, *HBA1c* Glycosylated hemoglobin, *DBEE* Dynamic back extensors endurance, *COPD* Chronic obstructive pulmonary disease, *QoL* Quality of life, *OA* Osteoarthritis, *DRPEE* Dynamic Back Endurance Exercise, *US* Ultrasound, *SQS* Static quadriceps muscle strength, *TOP* Transverse Oscillatory pressure, *ADL* Activity of daily living, *FAB* Fear-Avoidance Beliefs IQS- isometric quadriceps strengthening, *SWD* Short wave diathermy, *RM* Repetitive maximum, *RMDQ* Roland-Morris Disability Questionnaire, *FPG TENS* Transcutaneous electrical stimulation, *PID* Pelvic inflammatory disorder, *HTN* Hypertension, *RCT* Randomized controlled trial

### Study designs, sample size, & sampling techniques

Of the 76 studies included in this review, 64 (84.2%) and 12 (15.8%) were randomized control trials (Additional file 1: Appendix 3 Table 1) and non-randomized experiments (Additional file 1: Appendix 3 Table 2), respectively. Regarding sample size, Onigbinde et al. [88] and Ogbutor et a [74]. utilized the least (17) and largest (400) sample sizes, respectively. Probability sampling techniques were employed in 64 (84.2%) of the studies (RCTs), while non-probability techniques were utilized in 12 (15.8%) studies (non-randomized).

### Statistics and confounding bias

On assessment for the test of normality of dataset, 12 (15.8%) studies [25, 26, 32, 38, 40, 41, 52, 54, 63, 69, 70, 84, 96] performed a normality test. Eleven of them [22, 32, 38, 40, 41, 52, 54, 63, 69, 70, 84, 96] used the Shapiro–Wilk test while Abdulahi [38] employed Kolmogorov test. Besides the lack of normality test. Of the 76 included studies, 31 (42.1%) [25–27, 38, 42, 43, 45, 47, 52–55, 58, 61, 62, 65, 66, 72–74, 76, 77, 79–81, 88–91, 93, 97] suffered confounding-by-indication bias. Sources of confounding bias were missing baseline comparison of relevant socio-demographic characteristics 11 (14.5%) [38, 42, 43, 45, 52, 54, 58, 61, 65, 66, 89], missing baseline comparison of key outcome(s) 8 (10.5%) [25–27, 54, 72, 73, 80, 90], missing baseline comparison of both socio-demographic characteristics and key outcome(s)and failure to account for a statistically significant difference in baseline levels of relevant outcomes in the final post-intervention analysis 15 (19.7%). A few studies (11.8%) [22, 24, 38, 43, 47, 63, 69, 70, 98] employed an intention-treat approach to statistical analysis.

### Study power and effect size

The average study power was 72.3%. More than half (56.6%) of the studies were sufficiently powered, with 36 (21%) of them being overtly powered. This is a desirable outcome, however, 43.3% of the clinical experiments were underpowered. Regarding the magnitude of study effect, 12 (15.8%) studies recorded nil effects, 11 (14.5%) had small effects, 20 (26.3%) with medium effects, and 32 (42.1%) had large effects (Table 2). We observed incongruence between effect size and study power in 31 (40.8%) of the studies.

## Discussion

This review was conducted to characterize and critique physiotherapy interventional research in Nigeria. Of the clinical experiments, the randomized controlled trial (RCT) was the most common design. Musculoskeletal conditions were the most studied disorder. More than half of studies possessed fair to good quality. In the order of frequency, interventions constituted exercise therapy, manual therapy and electrotherapy. More than half of the studies recorded medium to large effect sizes. A fair proportion of the studies had a confounding-by-indication bias. A few studies conducted normality tests and intention-to-treat analysis. The fact that the majority of the studies were excluded for being non-interventional reflects a poor volume of interventional research compared with the expected volume of clinical trials from postgraduate Physiotherapy research, particularly PhDs, in the country. This suggests that clinical trials are an uncommon engagement among physiotherapist researchers in Nigeria. However, the number may reflect the volume of unpublished experimental theses and dissertations as well as the numbered published in fair to high impact journals indexed with Medline, Pubmed, and Cumulative Index for Nursing, Allied Health and Life Sciences, Academic Search Complete. This collaborates with the findings of Hamzat et al. [10] and Adeniyi et al. [11]. Hazmat [10] reported a low level of participation in clinical research among Nigerian physiotherapists, while Adeniyi et al. [11] reported cross-sectional design to be most prevalent study design among undergraduate students. Research output is a required criterion for the promotion of University academics in Nigeria. However, whether a study is a clinical experiment or a once-off observation study does not make a difference in the appraisal score for promotion of academics, thus disfavoring the chances for more rigorous and costly clinical experiments [99, 100].

Of the clinical experiments, randomized control trials were the most prevalent. This is a desirable outcome because RCTs form the gold standard design for responsible conduct of clinical research especially ones seeking the development of clinical intervention and innovation [101, 102]. Interestingly, the fact that only a few of the RCTs preserved the integrity of randomization through intention-to-treat analysis is undesirable. Randomization can greatly reduce the unintentional bias and confounding effects that may exist between the control group and the intervention group. Randomization ensures the later use of probability theory to perform the statistical analysis [103]. It is important to make sure the control and treatment have the same conditions in various aspects [104]. Contrary to this outcome of the study, Adeniyi et al. [11] found cross-sectional observational studies to be the most prevalent. However, the choice of cross-sectional designs over RCT designs may be due to the limited time allocated (i.e. having less than 6 months) for the completion of undergraduate research project.

We found a fair proportion of statistical analysis inappropriate in a fair proportion of the studies, with lack of covariate analysis and lack of normality test being the major reasons. This indicates a fair degree of inadequacy of statistical education among Physiotherapy researchers in Nigeria. This supports the advocacy for statistical education among Nigeria researchers [105], as knowledge of statistics is essential for biomedical research and innovation. Although this is the first methodical review appraising statistical application among Nigerian clinical research, the finding collaborates that of Yan et al. [104] Common statistical omissions or malpractices include ignoring the sample size and data distribution, incorrect summarization measurement, wrong statistical test methods especially for repeated measures, ignoring the assumption for t-test or ANOVA test, failing to perform the adjustment for multiple comparisons [104]. Confounds have been known to introduce bias in research studies [106]. In interventional research, the presence of significant bias raises the question of whether the outcomes are credible and recommended for application in the clinical setting for the management of patients and clients [104, 106]. Without the proper application of statistical methods, the risk of confounding bias increases, data collection suffers from additional costs and efforts and the analysis of research data gives suboptimal results and eventually, wrong decisions can be taken [105].

Interestingly, although most studies were of fair to good quality in terms of risk of bias assessment, the enormity of omissions in the included studies collaborates finding endorsed by the UK Department for International Development [12], which revealed a remarkable prevalence of low-quality research in Nigeria. Factors associated with poor-quality research in Nigeria include weak national research funding capacity, and lack of mechanisms for research quality evaluation [12]. Quality appraisal assessment is important in permitting researchers to draw effective conclusions and broader inferences concerning the primary study [107]. Furthermore, our study findings align with Hassan and Schaffer [108] which reported poor quality of clinical research and science in Africa. The lack of high-quality research in Nigeria constitutes an impediment to the growth and development of the country Garba and Saidu [14]. The fraction of resources imputed into research output is minimal and lack of funding, equipment and mentoring are the prominent reasons for the poor quality of research [14]. Therefore, addressing these putative barriers will improve the quality of physiotherapy intervention research in the Nigerian context and as well increase the chance of translation of clinical research outcomes into impactful products, decisions and policies [14, 109, 110].

More than half of the studies included studies were sufficiently powered, with 50% being overtly powered. This is a desirable outcome, however, over 43% of the clinical experiments are underpowered thereby resulting in an average statistical power of approximately 72%. This is undesirable and indicates the need for statistical consultation and/or inclusion of biostatisticians as staff in clinical departments across Nigerian academic institutions. This is because a low statistical power has a reduced chance of detecting a true effect and the chance that a statistically significant result reflects a true effect. Similarly, about half of the studies were overtly powered and this reflects wastefulness [111] in the conduct of clinical physiotherapy intervention research in Nigeria. Furthermore, possession of adequate sample size is a good trait of quality clinical research, however, the incongruence between study quality (mostly suboptimal) and power (mostly sufficient) in our review reflects a lack of statistical consultation and/or expertise in RCT among Physiotherapy researchers in Nigeria [105]. Given this incongruence, the findings that most of the studies observed medium to large effect cast doubt on the efficacy of Physiotherapy interventions as reported in the Nigerian studies, thus requiring further investigation.

Assessment of effect size otherwise known as the magnitude of treatment effects revealed that Physiotherapeutic interventions as utilized in this study are largely effective for the disorders indicated in the studies. This is consistent with results obtained by Herbert et al. [112] who found various physiotherapy interventions-exercise and massage yielded positive health outcomes in the patient population studied. Our review, show that exercise was the most important intervention or treatment modality employed among Nigerian Physiotherapists. This shows that exercise as a mainstay physiotherapy intervention is of significant benefits. This is in keeping with an evidence-based recommendation as enshrined in clinical practice guidelines for the management of low-back pain [113, 114]. Also, the findings that electrotherapy modalities (usually used for pain) possessed nil to medium effect (21, 53, 57) reflects clinical practices guidelines recommendations to avoid electrotherapy modalities for the management of an orthopaedic condition like low-back pain because they have found to be ineffective [113, 114]. Despite this finding, the incongruence between study power and magnitude of treatment effects in most of the studies included in this review constitutes a serious limitation thus limiting the inference that physiotherapy interventions were largely efficacious for the health conditions and outcomes studied. Hence, there is a need for the pursuit of excellence when conducting further clinical Physiotherapy research as this is the only way to make research count.

The enormity of research and statistical flaws observed in the included papers raises a question on education and entry-level of physiotherapists in and through research, as well as the structuring of research in the country to support the development of quality scientific production. While we did not factor data on educational qualification of the chief/corresponding authors, a recent studies shows that most of Nigerian Physiotherapy academics possessed at least Master’s degree [14]. Clearly, mostly, there is a lack of consistency in structure as well as focus with topics often picked at random, without succint, well-thought out and tenacious trajectory towards achieving substantial research products of clinical developmental significance. Contrary, researches should be targeted at problem solving and not just to gain promotion, and should be domesticated and contextualized to reflect the needs and resource of the society [115].

Overall, the fact that that we may not have included all eligible studies constitutes a limitation. However, we made efforts to search relevant databases. Although, there were several omissions observed in the concluded studies, the fact that most of the studies possessed moderate to large effects as well as fair to good quality is a strength and tends to validates the policy underlying the establishment of physiotherapy as a healthcare profession in the country. However, the exact efficacy of the various physiotherapy interventions x-rayed in this review should be interpreted in the light of enormity of others flaws not captured in the risk of bias assessment.

## Conclusions

Of clinical experiments, RCT occupies the top row in the Nigerian physiotherapy community. Most of the studies possessed fair to good quality. The most important flaws include improper computation of sample size, statistical analysis, absent intention-to-treat approach, among others. The magnitude of effects of Physiotherapy interventions varies from nil effect to large effect. Exercise appears as the most utilized and effective intervention while electrotherapeutic modalities appears as the least important. Continuous professional training in research and biostatistics will improve the quality of physiotherapy intervention research. We recommend early introduction of research and statistical education to future physiotherapists.

### Supplementary Information

**Additional file 1: Appendix I. **Pubmed Piloted Search Strategy.

**Additional file 2. **Sociodemographic characteristics of the participants.

**Additional file 3: Table 1. **RISK OF BIAS USING PEDRO. **Table 2.** RISK OF BIAS USING ROBIN-I.

## Data Availability

Review data is available from the corresponding author on request.

## References

[1] Ghaemi SN, Goodwin FK (2007). The ethics of clinical innovation in psychopharmacology: challenging traditional bioethics. Philos Ethics Humanit Med.

[2] Barbosa D. Importância da pesquisa clínica para a prática na área de saúde. Acta Paul Enferm. 2010;23(1):vii-. 10.1590/S0103-21002010000100001.

[3] Cascella M, Rajnik M, Aleem A, et al. Features, Evaluation, and Treatment of Coronavirus (COVID-19). In: StatPearls. Treasure Island (FL): StatPearls Publishing; 2023 Jan-. Available from: https://www.ncbi.nlm.nih.gov/books/NBK554776/4. Updated 2023 Jan 9. 32150360

[4] Gostin LO, Nass S (2009). Reforming the HIPAA privacy rule: safeguarding privacy and promoting research. J Am Med Assoc.

[5] Bates DW, Leape LL, Cullen DJ, Laird N, Petersen LA, Teich JM (1998). Effect of computerized physician order entry and a team intervention on prevention of serious medication errors. J Am Med Assoc.

[6] National Research Council. Advancing the Nation’s health needs: NIH research training programs. Washington, DC: National Academies Press; 2005. Available from: https://www.ncbi.nlm.nih.gov/books/NBK22631/. Accessed Jul 31, 2021. 20669451

[7] Food and Drug Administration. Center for Drug Evaluation and Research, Guidance for Industry, Investigators, and Institutional Review Boards. US Food and Drug Administration; 2020. Available from: https://www.fda.

[8] Balogun J, Balogun A, and Obajuluwa V. A phenomenological investigation of the early years of physiotherapy education in Nigeria. European J Physiother. 2017;4:1301183.

[9] Oke KI. The past, the present and the future of physiotherapy in Nigeria. Guest Lecture at University of Benin Student Association Health week. 2019.

[10] Hamzat TK, Odole AC, Amusat NT (2002). Participation level of Nigerian Physiotherapists in clinical research. J Nigerian Society Physiotherapists.

[11] Adeniyi AF, Ekechukwu NE, Umar L, Ogwumike OO (2013). Research profile of Physiotherapy undergraduates in Nigeria. Educ Health (Abingdon).

[12] Fosci M, Loffreda L, Chamberlain A, Naidoo N. Assessing the needs of the research system in Nigeria. Report for the SRIA programme. Available from: https://assets.publishing.service.gov.uk/media/5ef4ad5ee90e075c5cc9cc7e6/; 2019.

[13] Balogun JA, Aka P, Balogun AO, Obajuluwa VA (2017). A phenomenological investigation of the first two decades OF university-based Physiotherapy education in Nigeria. Cogent Med.

[14] Balogun JA, Mbada CE, Balogun AO, Bello AI, Okafor UC. Profile of physiotherapist educators in anglophone West African countries: A cross-sectional study. Int J Med Health Sci. 2016;3(9):99–109. 10.18488/journal.9/2016.3.9/9.9.99.109.

[15] Garba B, Saidu B. Biomedical research in Nigeria: realities and misconceptions. Future Sci OA. 2020;6(7):FSO475. 10.2144/fsoa-2019-0157. 10.2144/fsoa-2019-0157 PMC7421249 32802389

[16] Tricco AC, Lillie E, Zarin W, O’Brien KK, Colquhoun H, Levac D (2018). PRISMA extension for scoping reviews (PRISMA-ScR): checklist and explanation. Ann Intern Med.

[17] Cashin AG, McAuley JH (2020). Clinimetrics: physiotherapy evidence database (PEDro) scale. J Physiother.

[18] Maher CG, Sherrington C, Herbert RD, Moseley AM, Elkins M (2003). Reliability of the PEDro scale for rating quality of randomized controlled trials. Phys Ther.

[19] Sterne JA, Hernán MA, Reeves BC, Savović J, Berkman ND, Viswanathan M (2016). ROBINS-I: a tool for assessing risk of bias in non-randomised studies of interventions. BMJ.

[20] Borenstein M, Hedges LV, Higgins JPT, Rothstein HR. Introduction to meta-analysis. 2009 edition. West Sussex: Wiley; 2009. p. 25–35.

[21] Lenhard DW. Computation of different effect sizes like d, f, r and transformation of different effect sizes. https://www.psychometrica.de/effect_size.html. Accessed 12 Aug 2019.

[22] Abass AO, Alli AR, Olagbegi OM, Christie CJ, Bolarinde SO (2020). Effects of an eight-week LUMBAR Stabilization exercise programme on selected variables of patients with chronic low back pain. Bangladesh J Med Sci.

[23] Abdullahi A (2018). Effects of number of repetitions and number of hours of shaping practice during constraint-induced movement therapy: A randomized controlled trial. Neurol Res Int.

[24] Akinola BI, Gbiri CA, Odebiyi DO. Effect of a 10-week aquatic exercise training program on gross motor function in children with spastic cerebral palsy. Glob Pediatr Health. 2019;6:2333794X19857378. 10.1177/2333794X19857378. 10.1177/2333794X19857378 PMC6595635 31263742

[25] Adeniyi AF, Fasanmade AA, Sanya AO, Borodo M (2010). Neuromusculoskeletal disorders in patients with type 2 diabetes mellitus: outcome of a twelve-week therapeutic exercise programme. Niger J Clin Pract.

[26] Adeniyi AF, Uloko AE, Ogwumike OO, Sanya AO, Fasanmade AA. Time course of improvement of metabolic parameters after a 12 week physical exercise programme in patients with type 2 diabetes: the influence of gender in a Nigerian population. BioMed Res Int. 2013;2013:Article ID 310574. 10.1155/2013/310574. 10.1155/2013/310574 PMC3773397 24078913

[27] Adepoju F, Hamzat T, Akinyinka O (2017). Comparative efficacy of progressive resistance exercise and biomechanical ankle platform system on functional indices of children with cerebral palsy. Ethiop J Health Sci.

[28] Ahmed UA, Maharaj SS, Van Oosterwijck J (2021). Effects of dynamic stabilization exercises and muscle energy technique on selected biopsychosocial outcomes for patients with chronic non-specific low back pain: a double-blind randomized controlled trial. Scand J Pain.

[29] Ajiboye OA, Anigbogu CN, Ajuluchukwu JN, Jaja SI (2015). Exercise training improves functional walking capacity and activity level of Nigerians with chronic biventricular heart failure. HK Physiother J.

[30] Akodu AK, Akindutire OM (2018). The effect of stabilization exercise on pain-related disability, sleep disturbance, and psychological status of patients with non-specific chronic low back pain. Korean J Pain.

[31] Akodu AK, Nwanne CA, Fapojuwo OA (2021). Efficacy of neck stabilization and Pilates exercises on pain, sleep disturbance and kinesiophobia in patients with non-specific chronic neck pain: A randomized controlled trial. J Bodyw Mov Ther.

[32] Aliyu FY, Wasiu AA, Bello B (2018). Effects of a combined lumbar stabilization exercise and cognitive behavioral therapy on selected variables of individuals with non-specific low back pain: A randomized clinical trial. Fisioterapia.

[33] Asogwa EI, Abonyi OS, Elom CO, Oduma CA, Umoke CC, Ogai NA (2022). Comparative effects of 6-weeks progressive resistance exercise and moderate intensity aerobic exercise on CD4 count and weights of people living with HIV/AIDS in Alex-Ekwueme Federal University Teaching Hospital Ebonyi State. Med (Baltim).

[34] Aweto HA, Aiyegbusi AI, Ugonabo AJ, Adeyemo TA (2016). Effects of aerobic exercise on the pulmonary functions, respiratory symptoms and psychological status of people living with HIV. J Res Health Sci.

[35] Aweto HA, Obikeh EO, Tella BA (2020). Effects of incentive spirometry on cardiopulmonary parameters, functional capacity and glycemic control in patients with Type 2 diabetes. Hong Kong Physiother J.

[36] Aweto HA, Aiyegbusi AI, Olaniyan ZO (2017). A comparative study of the effects of incentive spirometry and diaphragmatic resistance training on selected cardiopulmonary parameters in patients with Asthma. Rom J Phys Ther.

[37] Bello B, Adeniyi AF (2018). Effects of lumbar stabilisation and treadmill exercise on function in patients with chronic mechanical low back pain. Int J Ther Rehabil.

[38] Bello B, Danazumi MS, Kaka B (2019). Comparative effectiveness of 2 manual therapy techniques in the management of lumbar radiculopathy: A randomized clinical trial. J Chiropr Med.

[39] Bolarinde SO, Adegoke B, Ayanniyi O, Olagbegi O (2017). Effects of stretching exercises on pain and functional disability in quarry workers with work-related low back pain. J Health Saf Res Pract.

[40] Danazumi MS, Ibrahim SU, Yakasai AM, Dermody G, Bello B, Kaka B (2021). A comparison between the effect of combined chain exercises plus Kinesio taping with combined chain exercises alone in knee osteoarthritis: A randomized clinical trial. Am J Phys Med Rehabil.

[41] Danazumi MS, Yakasai AM, Ibrahim AA, Shehu UT, Ibrahim SU (2021). Effect of integrated neuromuscular inhibition technique compared with positional release technique in the management of piriformis syndrome. J Osteopath Med.

[42] Ezema CI, Okwuchukwu CK, Amarachukwu CN, Nweke MC, Obiekwe C, Okafor CI (2019). Effect of a single bout interval aerobic exercise on blood glucose level in type 2 diabetes mellitus patients. Indian J Physiother Occup Ther.

[43] Ezema CI, Onwunali AA, Lamina S, Ezugwu UA, Amaeze AA, Nwankwo MJ (2014). Effect of aerobic exercise training on cardiovascular parameters and CD4 cell count of people living with human immunodeficiency virus/acquired immune deficiency syndrome: A randomized controlled trial. Niger J Clin Pract.

[44] Ezema CI, Omeh E, Onyeso OKK, Anyachukwu CC, Nwankwo MJ, Amaeze A (2019). The effect of an aerobic exercise programme on blood glucose level, cardiovascular parameters, peripheral oxygen saturation, and body mass index among Southern Nigerians with type 2 diabetes mellitus, undergoing concurrent sulfonylurea and metformin treatment. Malays J Med Sci.

[45] Fadupin GT, Akinola OA (2011). Importance of accurate food measurement and physical exercise on BMI and glycaemic control in obese type 2 diabetic patients. Afr J Diabetes Med.

[46] Fayehun AF, Olowookere OO, Ogunbode AM, Adetunji AA, Esan A (2018). Walking prescription of 10 000 steps per day in patients with type 2 diabetes mellitus: A randomised trial in Nigerian general practice. Br J Gen Pract.

[47] Habibu W, Hanif S (2017). Efficacy of non-thermal ultrasound in the management of breast engorgement in post-partum women: A randomized controlled trial. Af Jrl Phys Rehab Sci.

[48] Ibrahim AA, Akindele MO, Ganiyu SO (2023). Effectiveness of patient education plus motor control exercise versus patient education alone versus motor control exercise alone for rural community-dwelling adults with chronic low back pain: a randomised clinical trial. BMC Musculoskelet Disord.

[49] Idowu OA, Adeniyi AF (2020). Efficacy of graded activity with and without daily-monitored-walking on pain and back endurance among patients with concomitant low-back pain and Type-2 diabetes: A randomized trial. Ethiop J Health Sci.

[50] Ige OM, Olarewaju RK, Lasebikan VO, Adeniyi YO (2010). Outpatient pulmonary rehabilitation in severe chronic obstructive pulmonary disease. Indian J Chest Dis Allied Sci.

[51] Jegede JA, Adegoke BOA, Olagbegi OM (2017). Effects of a twelve-week weight reduction exercise programme on selected spatiotemporal gait parameters of obese individuals. J Obes.

[52] John DO, Tella BA, Olawale OA, John JN, Adeyemo TA, Okezue OC (2018). Effects of a 6-week aerobic exercise programme on the cardiovascular parameters, body composition, and quality of life of people living with human immune virus. J Exer Rehabil.

[53] Johnson OE, Adegoke BOA, Ogunlade SO (2010). Comparison of four physiotherapy regimens in the treatment of long-term mechanical low back pain. J Jpn Phys Ther Assoc.

[54] Kaka B, Ogwumike OO, Adeniyi AF, Maharaj SS, Ogunlade SO, Bello B (2018). Effectiveness of neck stabilisation and dynamic exercises on pain intensity, depression and anxiety among patients with non-specific neck pain: a randomized controlled trial. Scand J Pain.

[55] Lamina S, Okoye GC (2012). Therapeutic effects of a moderate intensity interval training program on the lipid profile in men with hypertension: A randomized controlled trial. Niger J Clin Pract.

[56] Lamina S, Okoye CG, Hanif SM (2013). Randomised controlled trial: effects of aerobic exercise training programme on indices of adiposity and metabolic markers in hypertension. J Pak Med Assoc.

[57] Lamina S, Okoye G, Anele T, Ezema C, Ezugwu A (2013). Effect of interval training program on rate-pressure product in the management of hypertension in black African male subjects: A randomized controlled trial. Niger J Basic Clin Sci.

[58] Lamina S, Okoye GC (2013). Effect of interval training programme on pulse pressure in the management of hypertension: A randomized controlled trial. Afr Health Sci.

[59] Lamina S, Hanif S, Gagarawa YS (2011). Short wave diathermy in the symptomatic management of chronic pelvic inflammatory disease pain: A randomized controlled trial. Physiother Res Int.

[60] Lamina L, Okoye GC (2014). Therapeutic effect of continuous exercise training program on serum creatinine concentration in men with hypertension: A randomized controlled trial. Ghana Med J.

[61] Maduagwu SM, Gashau W, Balami A, Kaidal A, Oyeyemi AY, Danue BA et al. Aerobic exercise improves quality of life and CD4 cell counts in HIV seropositives in Nigeria. J Hum Virol Retrovirol. 2017;5(3). 10.15406/jhvrv.2017.05.00151. 10.15406/jhvrv.2017.05.00151 PMC6433410 30918915

[62] Sm M, A K, W G, A B, Ac O, Ba D et al. Effect of aerobic exercise on CD4 cell count and lipid profile of HIV infected persons in North Eastern Nigeria. J AIDS Clin Res. 2015;6(10):508. 10.4172/2155-6113.1000508. 10.4172/2155-6113.1000508 PMC6433405 30918743

[63] Maharaj SS, Nuhu JM. Rebound exercise. A beneficial adjuvant for sedentary non-insulin-dependent type 2 diabetic individuals in a rural environment. Aust J Rural Health. 2016;24(2):123–9. 10.1111/ajr.12223. 10.1111/ajr.12223 26255814

[64] Maruf FA, Akinpelu AO, Salako BL (2014). A randomized controlled trial of the effects of aerobic dance training on blood lipids among individuals with hypertension on a thiazide. High Blood Press Cardiovasc Prev.

[65] Maruf FA, Akinpelu AO, Salako BL (2013). Effects of aerobic exercise and drug therapy on blood pressure and antihypertensive drugs: a randomized controlled trial. Afr Health Sci.

[66] Maruf FA, Akinpelu AO, Salako BL, Akinyemi JO (2016). Effects of aerobic exercise dance training on blood pressure in individuals with uncontrolled hypertension on two antihypertensive drugs: a randomized clinical trial. J Am Soc Hypertens.

[67] Mbada CE, Ayanniyi O, Ogunlade SO, Orimolade EA, Oladiran AB, Ogundele AO (2013). Rehabilitation of back extensor muscles’ inhibition in patients with long-term mechanical low-back pain. ISRN Rehabil.

[68] Mbada CE, Ayanniyi O, Ogunlade SO (2015). Comparative efficacy of three active treatment modules on psychosocial variables in patients with long-term mechanical low-back pain: a randomized-controlled trial. Arch Physiother.

[69] Nweke M, Nombeko M, Govender N, Aderonke Akinpelu O (2021). Physiologic effects of physical activity on cognitive function in people living with HIV: a systematic review of intervention and observational studies. Afr J Phys Act Health Sci (AJPHES).

[70] Nweke M, Nombeko M, Govender N, Akineplu AO (2022). Rehabilitation of HIV-associated neurocognitive disorder: a systematic scoping review of available interventions. Adv Ment Health.

[71] Odebiyi DO, Aborowa AT, Sokunbi OG, Aweto HA, Ajekigbe AT (2014). Effects of exercise and oedema massage on fatigue level and quality of life of female breast cancer patients. Eur J Physiother.

[72] Odole AC, Ojo OD (2013). A telephone-based physiotherapy intervention for patients with osteoarthritis of the knee. Int J Telerehabil.

[73] Odunaiya NA, Agbaje SA, Adegoke OM, Oguntibeju OO (2022). Effects of a four-week aerobic exercise programme on depression, anxiety and general self-efficacy in people living with HIV on highly active anti-retroviral therapy. AIDS Care.

[74] Ogbutor GU, Nwangwa EK, Uyagu DD (2019). Isometric handgrip exercise training attenuates blood pressure in prehypertensive subjects at 30% maximum voluntary contraction. Niger J Clin Pract.

[75] Ogwumike OO, Arowojolu AO, Sanya AO (2011). Effects of a12-week endurance exercise program on adiposity and flexibility of Nigerian perimenopausal and postmenopausal women. Niger J Physiol Sci.

[76] Ojeniweh ON, Ezema CI, Anekwu EM, Amaeze AA, Olowe OO, Okoye GC (2015). Efficacy of six weeks infrared radiation therapy on chronic low back pain and functional disability in national orthopaedic hospital, Enugu, South East, Nigeria. The Nigerian Health [journal].

[77] Ojoawo AO, Olabode AD (2018). Comparative effectiveness of transverse oscillatory pressure and cervical traction in the management of cervical radiculopathy: A randomized controlled study. Hong Kong Physiother J.

[78] Ojoawo AO, Olabode A, Esan O, Badru A, Odejide S, Arilewola B (2013). Therapeutic efficacy of cervical traction in the management of cervical radiculopathy: A control trial. Rwanda J Health Sci.

[79] Ojoawo AO, Hassan MA, Olaogun MOB, Johnson EO, Mbada CE (2017). Comparative effectiveness of two stabilization exercise positions on pain and functional disability of patients with low back pain. J Exer Rehabil.

[80] Ojoawo AO, Malomo EO, Olusegun EO, Olaogun BMO (2018). Effects of pulse ultrasound and kneading massage in managing individual with incessant pain at lower region of back using random allocation. J Exer Rehabil.

[81] Ojoawo AO, Olabode A, Esan O, Badru A, Odejide S, Arilewola B (2016). Transverse oscillatory pressure in management of cervical radiculopathy: A randomised controlled study. Hong Kong Physiother J.

[82] Ojoawo AO, Olaogun MO, Odejide SA, Badru AA (2013). Effect of vertical oscillatory pressure on disability of patients with chronic mechanical low back pain using Roland Morris Disability questionnaire. Tanzan J Health Res.

[83] Ojoawo AO, Olaogun MOB, Hassan MA (2016). Comparative effects of proprioceptive and isometric exercises on pain intensity and difficulty in patients with knee osteoarthritis: A randomised control study. Technol Health Care.

[84] Okonkwo UP, Ibeneme SC, Ihegihu EY, Egwuonwu AV, Ezema IC, Maruf AF (2018). Effects of transcutaneous electrical nerve stimulation in the Management of Post-Injection Sciatic Pain in a non-randomized controlled clinical trial in Nnewi, Nigeria. BMC Complement Altern Med.

[85] Okonkwo UP, Ibeneme SC, Ihegihu EY, Egwuonwu AV, Ezema CI, Maruf FA (2018). Effects of a 12-month task-specific balance training on the balance status of stroke survivors with and without cognitive impairments in Selected Hospitals in Nnewi, Anambra State. Nigeria Top Stroke Rehabil.

[86] Olagbegi OM, Adegoke BO, Odole AC (2017). Effectiveness of three modes of kinetic-chain exercises on quadriceps muscle strength and thigh girth among individuals with knee osteoarthritis. Arch Physiother.

[87] Olagbegi OM, Adegoke BOA, Odole AC (2016). Effectiveness of combined chain exercises on pain and function in patients with knee osteoarthritis. Bangladesh J Med Sci.

[88] Onigbinde AT, Awotidebe T, Awosika H (2009). Effect of 6 weeks wobble board exercises on static and dynamic balance of stroke survivors. Technol Health Care.

[89] Onigbinde AT, Ajiboye RA, Bada AI, Isaac SO (2017). Inter-limb effects of isometric quadriceps strengthening on untrained contra-lateral homologous muscle of patients with knee osteoarthritis. Technol Health Care.

[90] Onigbinde AT, Adebowale AC, Ojoawo AO, Sunday OA, Bosede A (2013). Comparative effects of pulsed and continuous short wave diathermy on pain and selected physiological parameters among subjects with chronic knee osteoarthritis. Technol Health Care.

[91] Onuwe HAK, Amadi K, Odeh SO (2013). Comparison of the therapeutic efficacy of double-modality therapy, phonophoresis and cryotherapy in the management of musculoskeletal injuries in adult Nigerian subjects. Niger J Physiol Sci.

[92] Onwunzo CN, Igwe SE, Umunnah JO, Uchenwoke CI, Ezugwu UA (2021). Effects of isometric strengthening exercises on pain and disability among patients with knee osteoarthritis. Cureus.

[93] Sarafadeen R, Ganiyu SO, Ibrahim AA (2020). Effects of spinal stabilization exercise with real-time ultrasound imaging biofeedback in individuals with chronic nonspecific low back pain: a pilot study. J Exerc Rehabil.

[94] Sokunbi G, Takai IU, Nwosu IB, Balarabe R (2020). Effects of acupressure and acupuncture-like transcutaneous electrical nerve stimulation on sleep quality among pregnant women. J Acupunct Meridian Stud.

[95] Tella BA, Oghumu SN, Gbiri CAO (2022). Efficacy of transcutaneous electrical nerve stimulation and interferential current on tactile acuity of individuals with nonspecific chronic low back pain. Neuromodulation.

[96] Usman Z, Maharaj SS, Kaka B (2019). Effects of combination therapy and infrared radiation on pain, physical function, and quality of life in subjects with knee osteoarthritis: A randomized controlled study. Hong Kong Physiother J.

[97] Lamina S, Okoye G (2012). Effects of aerobic exercise training on psychosocial status and serum uric acid in men with essential hypertension: A randomized controlled trial. Ann Med Health Sci Res.

[98] Abdullahi A, Aliyu NU, Useh U, Abba MA, Akindele MO, Truijen S (2021). Comparing two different modes of task practice during lower limb constraint-induced movement therapy in people with stroke: A randomized clinical trial. Neural Plast.

[99] Pluye P, Gagnon MP, Griffiths F, Johnson-Lafleur JA (2009). A Scoring system for appraising mixed methods research, and concomitantly appraising qualitative, quantitative and mixed methods primary studies in mixed studies reviews. Int J Nurs Stud.

[100] ‘Lee YK, Shin ES, Shim JY, Min KJ, Kim JM, Lee SH et al.. Developing a scoring guide for the Appraisal of Guidelines for Research and Evaluation II instrument in Korea: a modified Delphi consensus process. J Korean Med Sci. 2013;28(2):190–4. 10.3346/jkms.2013.28.2.190. 10.3346/jkms.2013.28.2.190 PMC3565128 23400114

[101] Zabor EC, Kaizer AM, Hobbs BP (2020). Randomised controlled trials. Chest.

[102] Lim CY, In J (2019). Randomization in clinical studies. Korean J Anesthesiol.

[103] Bajwa SJS (2015). Basics, common errors and essentials of statistical tools and techniques in anesthesiology research. J Anaesthesiol Clin Pharmacol.

[104] Yan F, Robert M, Li Y (2017). Statistical methods and common problems in medical or biomedical science research. Int J Physiol Pathophysiol Pharmacol.

[105] Adelodun OA, Awe OO (2013). Statistics education in Nigeria: A recent survey. J Educ Pract.

[106] Wallach JD, Serghiou S, Chu L, Egilman AC, Vasiliou V, Ross JS (2020). Evaluation of confounding in epidemiologic studies assessing alcohol consumption on the risk of ischemic heart disease. BMC Med Res Methodol.

[107] Protogerou C, Hagger MS (2018). A case for a study quality appraisal in survey studies in psychology. Front Psychol.

[108] Hassan MHA, Schaffer D (2001). The Third World Academy of Sciences: celebrating two decades of progress. Curr Sci.

[109] Okoduwa SIR, Abe JO, Samuel BI, Chris AO, Oladimeji RA, Idowu OO et al.. Attitudes, Perceptions, and Barriers to Research and Publishing Among Research and Teaching Staff in a Nigerian Research Institute. Front Res Metr Anal;3. 10.3389/frma.2018.00026

[110] Nwagwu W (2005). Mapping of landscape of biomedical literature in Nigeria since 1979. Learn Publ.

[111] Case LD, Ambrosius WT (2007). Power and sample size. Methods Mol Biol.

[112] Herbert RD, Maher CG, Moseley AM, Sherrington C (2001). Effective physiotherapy. BMJ.

[113] de Campos TF (2017). Low back pain and sciatica in over 16s: assessment and management NICE guideline (NG59). J Physiother.

[114] Staal JB, Hendriks EJM, Heijmans M, et al. KNGF clinical practice guidelines for physical therapy in patients with low back pain. Amersfoort: the royal Dutch society for physical therapy (KNGF); 2013.

[115] Editorial board (2017) ‘Nigerian universities as problem-solvers’ The Guardian; Oct 26 2017. Available from: https://guardian.ng/opinion/nigerian-universities-as-problem-solvers/. Cited 28/5/2023.

