# Supplementary material for: Characterization and critical appraisal of physiotherapy intervention research in Nigeria: a systematic review
Source: BMC Musculoskelet Disord. 2024 Jan 2;25:27. doi: 10.1186/s12891-023-06986-7 (PMC10763218; doi:10.1186/s12891-023-06986-7)
Supplement: Supplementary file 1 — Additional file 1: Appendix I. Pubmed Piloted Search Strategy. [file 12891_2023_6986_MOESM1_ESM.docx]

Appendix I: Pubmed Piloted Search Strategy

| Search terms | Database | Date of search | Filter | No. retrieved |
| --- | --- | --- | --- | --- |
| (modalities, physical therapy OR physiotherapy techniques OR physiotherapy specialty OR specialty, physiotherapy OR exercises OR manual therapies OR manual therapy OR cryotherapy OR cryotherapies OR rehabilitation OR electrotherapy OR thermotherapy[MeSH Terms]) AND (intervention OR experiment OR treatment[MeSH Terms])) AND (Nigeria or Federal republic of Nigeria[MeSH Terms]) | PubMed | 17/01/2021 | Year | 288 |
