# Supplementary material for: Characterization and critical appraisal of physiotherapy intervention research in Nigeria: a systematic review
Source: BMC Musculoskelet Disord. 2024 Jan 2;25:27. doi: 10.1186/s12891-023-06986-7 (PMC10763218; doi:10.1186/s12891-023-06986-7)
Supplement: Supplementary file 2 — Additional file 2. Sociodemographic characteristics of the participants. [file 12891_2023_6986_MOESM2_ESM.docx]

Supplementary file 1: Sociodemographic characteristics of the participants

| **S/N** | **Author(S)** | **Age (years)**  **Exp control** | | **Gender (% males)**  **Exp control** | | | **Level of education**  **Exp control** | | **Country** |
| --- | --- | --- | --- | --- | --- | --- | --- | --- | --- |
| 1. 1 | Abass et al.**^24^** | 48.15±9.02 | 53.1±7.91 | 6 (30) | | 5 (25) | nr | nr | Nigeria |
|  | Abdulahi et al.**^25^** | 57.21 ± 10.01 | 58.83 ± 10.57 | 26.16% | | 20% | nr | nr | Nigeria |
|  | Abdullahi et al.**^63^** | 50.2 ± 13.9 | 47.8 ± 14.7 | 12 (48%) | | 13 (52%) | nr | nr | Nigeria |
| 1. 3 | Adeniyi et al**.^37^** | 47.9 ± 9.93 | 30-64yrs | 15 (34.9) | | No CG | nr | nr | Nigeria |
| 1. 5 | Adeniyi et al.**^38^** | 49.6 ± 3.7 years | No cntl | 8 (27.6) | | No CG | nr | nr | Nigeria |
|  | Adepoju et al.**^39^** | 8.35 ±2.5 | No cntl | nr | | nr | nr | nr | Nigeria |
|  | Ahmed et al.**^65^** | 38.99±9.25 | 41.22±8.4 | nr | | nr | nr | nr | Nigeria |
|  | Ajiboye et al.**^66^** | 56.1 ± 2.0 | 51.5 2.6 | Not Reported | | Not Reported | nr | nr | Nigeria |
|  | Akinola et al.**^63^** | 4.93 ± 1.98 | 5.41 ± 2.85 | nr | | nr | nr | nr | Nigeria |
| 1. 10 | Akodu & Akindutire**^67^** | 50.08 ± 15.45 | No contl | 58.6 | | No CG | nr | nr | Nigeria |
|  | Akodu et al.**^68^** | 47.12 ± 9.47 | 44.93±6.26 | 42.9% | | 50% | nr | nr | Nigeria |
| 1. 11 | Aliyu et al**.^26^** | 44.26 ± 13.11 | 40.28 ± 11.80 | 9 (47.4%) | | 7 (38.9%) |  |  | Nigeria |
|  | Asogwa et al.**^69^** | Nr | nr | nr | | nr | nr | nr | Nigeria |
| 1. 12 | Aweto et al.**^70^** | 30.67 ± 5.83 | 32.07±5.36 | 30.3% but not specified for study arms | | | nr | nr | Nigeria |
|  | Aweto et al.**^71^** | 54.47 ± 9.77 | 55.76 14.56 | 46.7% | nr | | nr | nr | Nigeria |
|  | Aweto et al.**^72^** | 34.79 ± 15.91 | 30.14+ 9.65 | nr | nr | | nr | nr | Nigeria |
|  | Bello & Adeniyi**^73^** | 42.20 ± 12.91 | 46.60 ± 11.60 | nr | nr | | nr | nr | Nigeria |
| 1. 13 | Bello et al.**^27^** | 45.44 (8.84) | 49.51 (10.06) | nr | | nr | nr | nr | Nigeria |
| 1. 15 | Bolarinde et al.**^74^** | 35.17 ± 7.02 | 33.600 ± 6.678 | nr | | nr | nr | nr | Nigeria |
|  | Danazumi et al.**^28^** | 33.13±3.53 | No CG | 81.25 | | No CG | nr | nr | Nigeria |
|  | Danazumi et al.**^29^** | 52.15±5.72 | No CG | nr | | nr | nr | nr | Nigeria |
| 1. 16 | Ezema et al.**^40^** | 40.07±9.72 | 32.47±10.41 | nr | | nr | nr | nr | Nigeria |
| 1. 17 | Ezema et al.**^59^** | 51.7 (5.0%) | 51.4 (5.0%) | 11 (44.0%) | | 11 (44.0%) | nr | nr | Nigeria |
|  | Ezema et al.**^75^** | 53.20±8.17 | 58.80±6.60 | nr | | nr | nr | nr | Nigeria |
| 1. 18 | Fadupin & Akinola**^41^** | 48±7 | 49±3 | 9(50) | | 8(44) | nr | nr | Nigeria |
| 1. 19 | Fayehun et al.**^76^** | 16 were within 40–59  6 were within 60–64 | 40–59 16  60–64 6 | 35% | | 39% | Pry school 69.57%  Sec 30.43% | Pry school 21.74%  Sec 78.26% | Nigeria |
|  | Habibu & Hanif^42^ | nr | nr | nr | | nr | nr | nr | Nigeria |
| 1. 20 | Ibrahim et al.^77^ | 45.1±14.2 | 47.78±15.9 | 57.5 | | 65% | nr | Nr | Nigeria |
| 1. 22 | Idowu & Adeniyi**^78^** | 48.28(9.41) | 48.27 (9.56) | (32%) | | 38%) | Pry school 0%  Sec 100% | Pry school 24%  Sec 76% | Nigeria |
| 1. 23 | Ige et al.**^79^** | 66 ±7.4 | No contl | 66.7% | | No contl | nr | nr | Nigeria |
| 1. 24 | Jegede et al.**^80^** | 32.0 ± 8.26 | 29.32 ± 6.06 | Not Reported | | Not Reported | nr | nr | Nigeria |
| 1. 25 | John et al.**^30^** | 41± 7.8 | 40.9± 8 | 8 (32.0) | | 11 (40.7) | Secondary  100% | Secondary 100% | Nigeria |
| 1. 26 | Johnson et al.**^43^** | 45.3 ± 8.1 |  | 21 (39.6%) | |  | nr | nr | Nigeria |
|  | Kaka et al.**^31^** | 46.8±12.5 | No CG | 47.87 | | No CG | 84% had at least primary education | No CG | Nigeria |
|  | Lamina & Okoye**^44^** | nr | nr | nr | | nr | nr | nr | Nigeria |
| 1. 29 | Lamina & Okoye**^81^** | 58.40 ± 6.91 | 58.27 ± 6.24 | All male | | All male | nr | nr | Nigeria |
|  | Lamina & Okoye**^82^** | 58.63±7.22 | 58.27±6.24 | All male | | All male | nr | nr | Nigeria |
|  | Lamina & Okoye**^45^** | 58.63±7.22 | 58.27±6.24 | All male | | All male | nr | nr | Nigeria |
|  | Lamina et al.**^83^** | 58.90±7.35 | 58.27±6.24 years | 140 (57.1%) | | 105 (42.9%) | nr | nr | Nigeria |
|  | Lamina et al.**^84^** | 28.46±5.6 | 30.4±5.6 | nr | | nr | nr | nr | Nigeria |
| 1. 30 | Maduagwu et al.**^47^** | 40.84±10.05 | 39.38±10.03 | 31.2 | | 40.6% | Pry school 37.5%  Sec 81.25% | Pry school 59.34%  Sec 40.63% | Nigeria |
|  | Maduagwu et al.**^48^** | 40.84±10.05 | 39.38±10.03 | 56.3% | | 84.4% | nr | nr | Nigeria |
|  | Maharaj & Nuhu**^32^** | 40.5 ± 6.50 | 38.5 ± 5.77 | 51.1 | | 53.3 | nr | nr | Nigeria |
| 1. 31 | Maruf et al.**^85^** | 50.38±8.39 | 52.32±8.06 | nr | | nr | nr | nr | Nigeria |
|  | Maruf et al.**^49^** | 50.8±8.31 | 54.75±8.56 | nr | | nr | nr | nr | Nigeria |
|  | Maruf et al.**^60^** | 50.80±8.31 | 54.75±8.56 | 13 (21.7) | | 22 (36.7) | nr | nr | Nigeria |
| 1. 32 | Mbada et al.**^86^** | 51.9 ± 7.36 | - | nr | | nr | nr | nr | Nigeria |
|  | Mbada et al.**^87^** | 51.9 ± 7.36 | - | nr | | nr | nr | nr | Nigeria |
|  | Nweke et al.**^33^** | nr | nr | nr | | nr | nr | nr | Nigeria |
|  | Nweke et al.**^34^** | 45.86±9.26 | 46.17±8.97 | nr | | nr | nr | nr | Nigeria |
| 1. 33 | Odebiyi et al.**^88^** | 46.00 8.40 | 54.00 14.00 | All female | | All female | nr | nr | Nigeria |
| 1. 34 | Odole & Ojo**^50^** | 56.04 ± 7.40 | 54.96 ± 7.81 | 56% | | 48% | nr | nr | Nigeria |
|  | Odunaiya et al.**^51^** | nr | nr | 41.5% but not reported per study arms | | | nr | nr | Nigeria |
| 1. 35 | Ogbutor et al.**^23^** | 40.78±6.04 | 41.27±6.31 | 54.5% | | (56%) | nr | nr | Nigeria |
| 1. 36 | Ogwumike et al.**^89^** | 52.7 ± 3.95 | 51.7 ± 54.3 | All female | | All female | nr | nr | Nigeria |
|  | Ojeniweh et al**.^52^** | 50.04 ± 8. 79 | 40.0± 10.24 | nr | | nr | nr | nr | Nigeria |
|  | Ojoawo & Olabode**^53^** | 53.5±5.95 | 59.50 ± 2.64 | 58% | | 44% | nr | nr | Nigeria |
|  | Ojoawo et al.**^90^** | 51.38 + 6.5 | 59.50 + 2.64 | nr | | nr | nr | nr | Nigeria |
| 1. 37 | Ojoawo et al.**^54^** | 51.70±11.57 | nr | nr | | nr | nr | nr | Nigeria |
| 1. 38 | Ojoawo et al.**^55^** | nr | nr | nr | | nr | nr | nr | Nigeria |
| 1. 39 | Ojoawo et al.**^56^** | 55.67 5.35 | 59.50 2.646 | 61.5% | | 46.1% | nr | nr | Nigeria |
|  | Ojoawo et al.**^91^** | 53.37 10.84 but not specific for study arm | | nr | | nr | nr | nr |  |
| 1. 40 | Ojoawo et al.**^92^** | 51.38 6.545 55.67 5.35 | 59.50 2.646 | 60% | | 44% | nr | nr | Nigeria |
| 1. 41 | Okonkwo et al.**^35^** | 29.737 ± 15.225 | 38.409 ± 18.157 | 60% | | 50% | nr | nr | Nigeria |
|  | Okonkwo et al.**^93^** | 53.94 9.3 | 49.30 ± 12.2 | 46% | | 44% | nr | nr | Nigeria |
| 1. 43 | Olagbegi et al**.^94^** | 61.1±10.41 | NR | 38.6% but not specified for the study arms | | | nr | nr | Nigeria |
|  | Olagbegi et al.**^95^** | 61.27± 13.73 | - | nr | nr | | nr | nr | Nigeria |
|  | Onigbinde et al.**^22^** | 42–61 | 44–66 | nr | nr | | nr | nr | Nigeria |
|  | Onigbinde et al**.^61^** | 64 ±12.53 but no specific for the study arms | | 9% but no specific for the study arms | | | nr | nr | Nigeria |
|  | Onigbinde et al.**^62^** | 56.89±9.15 | No CG | nr |  | | nr | nr | Nigeria |
| 1. 44 | Onuwe et al**.^57^** | 34 (18-77) | No ctrl | 71.2 | | No ctrl | nr | nr | Nigeria |
| 1. 45 | Onwunzo et al.**^96^** | 60.30 ± 7.58 | 56.70 ± 7.66 | 25%) | | 20% | nr | nr | Nigeria |
| 1. 46 | Sarafadeen et al.**^58^** | 49.6± 22.3 but no specific for the study arms | | 50% but no specific for the study arms | | | nr | nr | Nigeria |
|  | Sokunbi et al.**^97^** | 26.35 ±6.36 | 28.0±9.52 | All female | | All female | nr | nr | Nigeria |
|  | Tella et al.**^98^** | 49.94 ±11.73 | - | nr | | nr | nr | nr | Nigeria |
| 1. 47 | Usman et al.**^36^** | 65.8 ±9.21 | 66.8 ±8.61 | 20 | | 40 | nr | nr |  |
