# Supplementary material for: Characterization and critical appraisal of physiotherapy intervention research in Nigeria: a systematic review
Source: BMC Musculoskelet Disord. 2024 Jan 2;25:27. doi: 10.1186/s12891-023-06986-7 (PMC10763218; doi:10.1186/s12891-023-06986-7)
Supplement: Supplementary file 3 — Additional file 3: Table 1. RISK OF BIAS USING PEDRO. Table 2. RISK OF BIAS USING ROBIN-I. [file 12891_2023_6986_MOESM3_ESM.docx]

Supplementary file 3 table 1: RISK OF BIAS USING PEDRO

| Authors | Q1 | Q2 | Q3 | Q4 | Q5 | Q6 | Q7 | Q8 | Q9 | Q10 | Q11 | TOTAL  SCORE | Quality Status |
| --- | --- | --- | --- | --- | --- | --- | --- | --- | --- | --- | --- | --- | --- |
| Abass et al. 2020 | YES | NO | NR | NO | NR | NR | NR | YES | YES | YES | YES | 5 | Fair |
| Akinola et al. 2019 | YES | YES | NR | YES | NO | NO | YES | YES | YES | YES | YES | 8 | Good |
| Abdulahi, 2018 | YES | YES | YES | YES | YES | NR | YES | YES | YES | YES | YES | 10 | Excellent |
| Adepoju et al. 2017 | YES | YES | NR | YES | NR | NO | YES | YES | YES | YES | YES | 8 | Good |
| Ajiboye et al. 2015 | YES | YES | YES | YES | NO | NO | NR | NO | NO | YES | YES | 6 | Good |
| Aliyu et al. 2018 | YES | YES | NO | YES | NO | NO | YES | YES | NO | YES | YES | 7 | Good |
| Aweto et al. 2016 | YES | YES | YES | YES | NO | NO | NR | YES | NO | YES | YES | 7 | Good |
| Bello et al. 2019 | YES | YES | YES | YES | NO | NO | NO | YES | YES | YES | NO | 7 | Good |
| Bello & Adeniyi 2018 | YES | YES | YES | YES | YES | NO | YES | YES | YES | YES | YES | 10 | Excellent |
| Bolarinde et al. 2017 | YES | YES | NR | YES | NO | NO | NO | YES | YES | YES | NO | 6 | Good |
| Ezema et al. 2014 | YES | NO | YES | YES | NO | NO | NO | YES | YES | YES | YES | 7 | Good |
| Ezema et al. 2019 | YES | YES | NR | NO | NO | NO | NR | YES | YES | YES | YES | 6 | Good |
| Fadupin & Akinola 2011 | YES | YES | NR | NO | NO | NO | NR | YES | YES | YES | YES | 6 | Good |
| Fayehun et al. 2018 | YES | YES | YES | YES | YES | NO | NO | YES | YES | YES | YES | 9 | Excellent |
| Aweto et al. 2020 | YES | YES | NR | YES | NO | NO | NR | YES | YES | YES | YES | 7 | Good |
| Idowu & Adeniyi 2020 | YES | YES | YES | YES | NO | NO | NR | YES | NO | YES | YES | 7 | Good |
| John et al. 2018 | YES | YES | NR | YES | NO | NO | NR | YES | YES | YES | NO | 6 | Good |
| Johnson et al. 2009 | YES | YES | NR | YES | NO | NO | YES | NO | NO | YES | YES | 6 | Good |
| Lamina et al. 2013 | YES | YES | NR | YES | NO | NO | NR | NO | YES | YES | YES | 6 | Good |
| Lamina & Okoye 2016 | YES | YES | NR | YES | NO | NO | NR | NO | NO | YES | YES | 5 | Fair |
| Lamina & Okoye 2011 | YES | YES | NR | YES | NO | NO | NR | NO | NO | YES | YES | 5 | Fair |
| Maduagwu et al. 2019 | YES | YES | NO | YES | NO | NO | NO | NO | NO | YES | NO | 4 | Fair |
| Maruf et al. 2013 | YES | YES | YES | YES | NO | YES | YES | NO | YES | YES | YES | 9 | Excellent |
| Mbada et al. 2013 | YES | YES | NR | YES | NO | NO | NO | NO | NO | YES | YES | 5 | Fair |
| Odebiyi et al. 2014 | YES | YES | NR | YES | NO | NO | NO | NO | NO | YES | YES | 5 | Fair |
| Odole & Ojo 2013 | YES | YES | NR | YES | NO | NO | NO | YES | YES | YES | NO | 6 | Good |
| Ogbutor et al. 2019 | YES | YES | YES | NR | NO | NO | NO | YES | YES | YES | YES | 7 | Good |
| Ogwumike et al. 2011 | YES | YES | NR | YES | NO | NO | NO | NO | YES | YES | YES | 6 | Good |
| Ojoawo et al. 2017 | YES | YES | NR | YES | NO | NO | NO | YES | NO | YES | NO | 5 | Fair |
| Ojoawo et al. 2018 | YES | YES | YES | NR | NO | NO | NO | NO | YES | YES | YES | 6 | Good |
| Ojoawo et al. 2016a | YES | YES | YES | YES | NO | NO | NO | NO | YES | YES | YES | 8 | Good |
| Ojoawo et al 2016b | NO | YES | YES | YES | NO | NO | NO | YES | YES | YES | YES | 7 | Good |
| Olagbegi et al. 2017 | YES | YES | YES | YES | YES | NO | NO | NO | YES | YES | YES | 8 | Good |
| Olagbegi et al. 2016 | YES | YES | YES | YES | NO | NO | NO | NO | NO | YES | NO | 5 | Fair |
| Onuwe et al. 2013 | YES | YES | YES | YES | NO | YES | YES | NO | YES | YES | NO | 8 | Good |
| Sikiru & Okoye 2014 | YES | YES | NO | YES | NO | NO | NO | NO | NO | YES | NO | 4 | Fair |
| Maduagwu et al. 2015 | YES | YES | NO | YES | NO | NO | NO | NO | NO | YES | NO | 4 | Fair |
| Usman et al. 2019 | YES | YES | YES | YES | NO | NO | NO | YES | YES | YES | YES | 8 | Good |
| Mbada et al. 2015 | YES | YES | YES | YES | NO | NO | NO | NO | NO | YES | NO | 5 | Fair |
| Sikiru & Okoye 2013 | YES | YES | NO | YES | NO | NO | NO | NO | NO | YES | YES | 5 | Fair |
| Kaka et al. 2018 | YES | YES | YES | YES | YES | NO | YES | YES | NO | YES | YES | 9 | Excellent |
| Maharaj et al. 2016 | YES | YES | NO | YES | NO | NO | YES | YES | YES | YES | YES | 8 | Good |
| Maruf et al. 2016 | YES | YES | YES | YES | NO | NO | YES | NO | YES | YES | YES | 8 | Good |
| Ojoawo et al. 2016 | YES | YES | YES | YES | NO | NO | NO | YES | NO | YES | YES | 7 | Good |
| Onigbinde et al. 2009 | YES | YES | NR | NO | NO | NO | NO | YES | YES | YES | NO | 5 | Fair |
| Teslim et al. 2013 | YES | YES | NR | YES | YES | NO | NO | YES | YES | YES | NO | 7 | Good |
| Onigbinde & Mustapha 2010 | YES | YES | NR | NO | NO | NO | NO | YES | NO | YES | NO | 4 | Fair |
| Ezema et al. 2019 | YES | YES | NR | NO | NO | NO | NO | YES | YES | YES | NO | 5 | Fair |
| Aweto et al. 2017 | YES | YES | NR | YES | NO | NO | NO | YES | YES | YES | YES | 7 | Good |
| Abdullahi et al. 2021 | YES | YES | YES | YES | YES | NO | YES | YES | YES | YES | NO | 9 | Excellent |
| Ahmed et al. 2021 | YES | YES | YES | YES | YES | NO | YES | YES | YES | YES | YES | 10 | Excellent |
| Akodu et al. 2020 | YES | YES | YES | YES | YES | NO | NR | YES | YES | YES | NO | 8 | Good |
| Danazumi et al. 2021a | YES | YES | YES | YES | NR | NR | NR | YES | YES | YES | YES | 8 | Good |
| Danazumi et al. 2021b | YES | YES | YES | YES | NO | NO | YES | YES | YES | YES | NO | 8 | Good |
| Habibu & Hanif, 2017 | YES | YES | YES | YES | NR | NO | NO | YES | YES | YES | YES | 8 | Good |
| Ibrahim et al. 2023 | YES | YES | YES | YES | YES | NO | YES | NO | YES | YES | YES | 9 | Excellent |
| Lamina et al. 2010 | YES | YES | YES | YES | NR | NR | NR | NO | NO | YES | YES | 6 | Good |
| Maruf et al. 2014 | YES | YES | NR | YES | NO | NO | NO | NO | YES | YES | YES | 6 | Good |
| Nweke et al. 2022a | YES | YES | YES | NO | NO | NO | YES | YES | YES | YES | YES | 8 | Good |
| Nweke et al. 2022b | YES | YES | YES | YES | NO | NO | YES | YES | YES | YES | YES | 8 | Good |
| Ojoawo et al. 2013 | YES | YES | NR | YES | NR | NR | NR | YES | YES | YES | YES | 7 | Good |
| Onwunzo et al. 2021 | YES | YES | NR | YES | NO | NO | NO | YES | YES | YES | YES | 7 | Good |
| Sokunbi et al. 2020 | YES | YES | YES | YES | NO | NO | YES | YES | YES | YES | YES | 9 | Excellent |
| Tella et al. 2021 | YES | YES | NR | YES | YES | NO | NO | NO | YES | YES | YES | 7 | Good |
| Q1. Subjects were randomly allocated to groups (in a crossover study, subjects were randomly allocated an order in which treatments were received); Q2. Allocation was concealed; Q3. The groups were similar at baseline regarding the most important prognostic indicators; Q4. There was blinding of all subjects; Q5. There was blinding of all therapists who administered the therapy; Q6. There was blinding of all assessors who measured at least one key outcome; Q7. Measures of at least one key outcome were obtained from more than 85% of the subjects initially allocated to groups; Q8. All subjects for whom outcome measures were available received the treatment or control condition as allocated or, where this was not the case, data for at least one key outcome was analysed by “intention to treat”; Q9. The results of between-group statistical comparisons are reported for at least one key outcome; Q10. The study provides both point measures and measures of variability for at least one key outcome.  NR= Not Reported | | | | | | | | | | | | |  |

Supplementary file 3 table 2: RISK OF BIAS USING ROBIN-I

| Authors | Bias due to cofounding | Bias due to selection of participants into the study | Bias due to classification of intervention | Bias due to deviations from intended intervention | Bias due to missing data | Bias in measurement of outcomes | Bias in selection of the reported result | Risk of Bias  status |
| --- | --- | --- | --- | --- | --- | --- | --- | --- |
| Adeniyi et al. 2013 | LOW | LOW | LOW | LOW | LOW | LOW | LOW | LOW RoB |
| Ige et al. 2010 | LOW | LOW | LOW | LOW | LOW | LOW | LOW | LOW RoB |
| Okonkwo et al. 2018 | LOW | LOW | LOW | LOW | LOW | LOW | LOW | LOW RoB |
| Onigbinde et al. 2016 | LOW | LOW | LOW | LOW | LOW | LOW | LOW | LOW RoB |
| Adeniyi et al. 2010 | LOW | LOW | LOW | LOW | LOW | LOW | LOW | LOW RoB |
| Akodu & Akindutire 2018 | LOW | LOW | LOW | LOW | LOW | LOW | LOW | LOW RoB |
| Ojoawo et al. 2013 | LOW | LOW | LOW | LOW | LOW | LOW | LOW | LOW RoB |
| Ojeniweh et al. 2015 | LOW | LOW | LOW | LOW | MODERATE | LOW | LOW | Moderate RoB |
| Okonkwo et al. 2018b | LOW | LOW | LOW | LOW | MODERATE | LOW | LOW | Moderate RoB |
| Sarafadeen et al. 2020 | LOW | LOW | LOW | LOW | LOW | LOW | LOW | LOW RoB |
| Odunaiya et al. 2021 | LOW | LOW | LOW | LOW | LOW | LOW | LOW | LOW RoB |
| Asogwa et al. 2022 | LOW | LOW | LOW | LOW | LOW | LOW | LOW | LOW RoB |
| RoB = Risk of Bias | | | | | | | | |
